# Liver and Hepatocyte Transplantation: What Can Pigs Contribute?

**DOI:** 10.3389/fimmu.2021.802692

**Published:** 2022-01-14

**Authors:** Xiaoxue Li, Ying Wang, Haiyuan Yang, Yifan Dai

**Affiliations:** ^1^ Jiangsu Key Laboratory of Xenotransplantation, Nanjing Medical University, Nanjing, China; ^2^ Key Laboratory of Targeted Intervention of Cardiovascular Disease, Collaborative Innovation Center for Cardiovascular Disease Translational Medicine, Nanjing Medical University, Nanjing, China; ^3^ State Key Laboratory of Reproductive Medicine, Nanjing Medical University, Nanjing, China; ^4^ Shenzhen Xenotransplantation Medical Engineering Research and Development Center, Institute of Translational Medicine, Shenzhen Second People’s Hospital, First Affiliated Hospital of Shenzhen University, Shenzhen, China

**Keywords:** coagulation disorders, hepatocyte xenotransplantation, hyperacute rejection, liver xenotransplantation, thrombocytopenia

## Abstract

About one-fifth of the population suffers from liver diseases in China, meaning that liver disorders are prominent causative factors relating to the Chinese mortality rate. For patients with end-stage liver diseases such as hepatocellular carcinoma or acute liver diseases with life-threatening liver dysfunction, allogeneic liver transplantation is the only life-saving treatment. Hepatocyte transplantation is a promising alternative for patients with acute liver failure or those considered high risk for major surgery, particularly for the bridge-to-transplant period. However, the lack of donors has become a serious global problem. The clinical application of porcine xenogeneic livers and hepatocytes remains a potential solution to alleviate the donor shortage. Pig grafts of xenotransplantation play roles in providing liver support in recipients, together with the occurrence of rejection, thrombocytopenia, and blood coagulation dysfunction. In this review, we present an overview of the development, potential therapeutic impact, and remaining barriers in the clinical application of pig liver and hepatocyte xenotransplantation to humans and non-human primates. Donor pigs with optimized genetic modification combinations and highly effective immunosuppressive regimens should be further explored to improve the outcomes of xenogeneic liver and hepatocyte transplantation.

## Introduction

With the rise in affluence, China has experienced a surge in the prevalence of liver disease, and the population effects have significant implications for global health (1). It is estimated that over one-fifth of the population in China is affected by some form of liver disease, notably hepatitis B virus, liver cirrhosis, liver cancer, and non-alcoholic fatty liver disease, making liver diseases one of the most significant contributions to health problems of the populace (2). Allogeneic liver transplantation is a life-saving treatment for end-stage liver diseases, hepatocellular carcinoma, or acute liver disease with life-threatening liver dysfunction (3–5). Liver transplantation can restore patients’ normal health and lifestyle and extend their lifespan by 15 years (6). According to the data from the Scientific Registry of Transplant Recipients, the overall survival rate of patients reached 90% one year after the death of a donor and 77% within five years (7). At present, the severe imbalance between liver supply and demand has become the main bottleneck restricting the application of liver transplantation. In the past ten years, the number of liver transplants in the United States has increased by 31% (8). More than 51,000 patients have died while waiting for liver transplants in the last 20 years (9).

To address the issue of organ supply, the clinical application of porcine xenogeneic livers remains an attractive goal. In addition to the advantages of physiological indicators and fewer ethical issues (10–12), considerable knowledge and experience have been gained regarding tissue typing and genetic engineering, making pigs more likely to become optimal donors (13). At the same time, the porcine endogenous retrovirus (PERV), which is the core of the pathogen’s cross-species infection, has also been knocked out in donor pigs, and the breeding of PERV knockout pigs has been realized (14, 15). The genetically modified donor pigs have achieved rapid, efficient, and diversified growth (16). The survival time of such pigs’ organs in non-human primates (NHPs) has been continuously extended. The longest survival time of recipients after orthotopic renal transplantation was 499 days (17), while the survival time of ectopic and orthotopic heart transplantation has reached up to 945 days and 195 days, respectively (18, 19).

As acute liver failure caused by chemical or viral hepatitis often has a sudden onset, it is usually impossible to determine a suitable donor organ before severe coagulopathy and/or death occurs (10). Due to the risk of major surgery in patients with severe liver diseases, alternative methods are needed to support liver function and regenerate the injured liver for those waiting for liver transplantation (20). Human hepatocyte transplantation has been successfully used in patients with acute liver failure and metabolic liver diseases (21–26). Meanwhile, porcine hepatocyte xenotransplantation has shown significant improvement of clinical parameters in small animal models of acute or chronic liver failure (27, 28). Since porcine hepatocyte xenotransplantation is much less invasive and allows the use of encapsulated and/or genetically modified cells, it provides an innovative approach to temporarily supporting liver function (29).

This brief review will summarize the development and potential therapeutic impact of pig liver and hepatocyte xenotransplantation in humans and NHPs as well as the remaining barriers that currently prevent widespread clinical application, including rejection, thrombocytopenia, and blood coagulation dysfunction.

## 1 Pig Liver Xenotransplantation

### 1.1 Wild-Type Pig-to-Human and -NHP Liver Xenotransplantation

Makowa et al. performed auxiliary liver transplantation in 1993 for a grade 3–4 hepatic coma patient with fulminant liver failure caused by autoimmune hepatitis using a wild-type (genetically unmodified) pig liver (30). Pre-treatment of the patient *via* the plasma elution method and specific antibody removal before the operation eliminated 90% of the natural anti-pig antibodies in the circulating blood of the recipient. The liver xenograft functioned, as shown by bile production and stabilization of prothrombin levels, and the liver function indicators tended to expected values after transplantation. In the end, the donor liver survived for 20 hours, and the recipient survived for 34 hours, demonstrating the ability of a pig liver to function at least temporarily. The autopsy revealed antibody-mediated immune rejection in the liver, suggesting that the heterogeneous antibodies in the circulating blood of the recipient could increase rapidly after being cleared. After the initial clinical failure of xenotransplantation studies using pigs as donors, the focus shifted to the field of basic research to find a way to eliminate rejection.

The first problem faced when a wild-type pig organ is transplanted into a patient or an NHP is the hyperacute rejection (HAR) associated with the binding of primate preformed antibodies to pig antigens. HAR leads to intravascular thrombosis, hemorrhagic necrosis, endothelial cell injury, and deposition of IgM, IgG, and C3 (10). For example, the first study in experimental unmodified pig-to-NHP liver xenotransplantation (LXT) was performed by Calne et al. in 1968 (31). Four of the seven baboons died of uncontrollable bleeding 6–30 hours after surgery. The longest survival time for the remaining recipients was only 3.5 days. Pathological examination showed that a large number of immune-inflammatory cells infiltrated the portal area. Subsequently, researchers used rhesus monkeys and gorillas as recipients; the animals survived less than 12 hours. During the next 20 years, the recipients’ survival time never exceeded three days, and thus the LXT studies at this stage all failed with wild-type pigs as donors. Therefore, genetically modified pigs were produced with the intention of alleviating rejection.

### 1.2 Genetically Modified Pig-to-NHP Liver Xenotransplantation

#### 1.2.1 Orthotopic Liver Xenotransplantation

The incidence of HAR was lowered when using organs from pigs expressing one or more human complement regulatory proteins (hCRPs) such as CD46, CD55, or CD59 (32) as the hCRPs play roles in repressing the recipients’ complement activation. In 2000, Ramirez et al. implemented the first orthotopic xenogeneic liver transplantation in baboons using genetically modified pigs expressing CD55 as donors (33). Neither liver xenograft demonstrated histopathological features of HAR. The baboons survived up to eight days. After that, medical researchers used livers from transgenic pigs with CD55, CD59, and H-transferase (34). Albeit the transplanted livers did not show obvious HAR and maintained a complete liver structure, the recipients experienced thrombosis, and survival time was only 13–24 h.

For HAR, the main target antigen is galactose-α-1,3-galactose, an oligosaccharide not present in humans, apes, or Old World monkeys (35, 36). Cooper et al. and Ekser et al. used α-1,3-galactosyltransferase gene-knockout (GTKO) minipigs expressing CD46(GTKO/hCD46) as donors to perform LXT (37, 38). The transplanted liver showed normal liver function according to relevant indicators in peripheral blood after the operation. However, the recipients experienced severe thrombocytopenia, leading to eventual death from abdominal bleeding and limiting recipient survival to a maximum of seven days. For severe thrombocytopenia after LXT, Kim et al. performed postoperative Amicar treatment of the recipients to inhibit fibrinolysis and prolonged the survival time of the recipients to nine days (39). Although the recipients did not develop fatal thrombocytopenia, they still suffered severe blood loss and sepsis. Navarro-Alvarez et al. supplemented human coagulation factors to recipients; however, these factors had no positive effect on prolonging survival (40). Baboons quickly developed extensive vessel thrombosis and thrombotic microangiopathy. Persistent thrombocytopenia, porcine graft thrombotic microangiopathy (TMA), and consumptive coagulopathy have been considered to be due to interspecies incompatibilities between pigs and NHPs (40).

Shah et al. documented 25 and 29-day survival of pCMV-free GTKO pigs to baboons treated with administration of exogenous human coagulation factors along with costimulation blockade (41, 42). Immunosuppressive therapy of recipients included induction with anti-thymocyte globulin, KF-506, methylprednisolone, and costimulation blockade (belatacept and anti-CD40 mAb, respectively). Thrombocytopenia spontaneously recovered within a few days after surgery without the need for platelet transfusions to recipients. The autopsies showed that the baboons had no evidence of rejection, inflammation, or TMA. These results recorded the longest survival time of pig-to-NHP LXT to date and represented an advance towards realistic consideration of the clinical applicability of LXT as a bridge to allotransplantation for patients.

#### 1.2.2 Heterotopic Liver Xenotransplantation

In 2013, Dou’s group transplanted a part of the liver from GTKO pigs as an auxiliary graft into Tibetan monkeys. Although the recipients required native splenectomy, none of the native liver needed to be excised, as the graft fitted comfortably into the splenic fossa. In theory, this can help control postoperative immune rejection and promote the recovery of the recipient’s liver function and help maintain normal coagulation and anti-coagulation functions (43, 44). The grafted liver functioned, as manifested by the regular physiological and biochemical indicators, common coagulation system, and stable platelet numbers. Monkeys showed no HAR or severe acute rejection after the operation, and survival time reached up to 14 days.

Yeh et al. at Massachusetts General Hospital of the United States also conducted a heterotopic auxiliary liver transplantation trial. After the operation, the recipients were supplemented with exogenous full-spectrum coagulation factors. The transplanted liver and the recipient survived for 15 days, and the recipient eventually died of liver failure and infection (45).

The most recent report documented a 26-day survival of a 13-gene modified pig liver graft in a rhesus monkey in 2021. The specific genotype of the donor pig is *PERV*-KO/*GalT*-KO/*β4GalNT*2-KO/*CMAH*-KO/*hCD46*/*hCD55*/*hCD59*/*hβ2M*/*hHLA-E*/*hCD47*/*hTHBD*/*hTFPI*/*hCD39* (*PERV*-KO/3-KO/9-TG), corresponding to the most extensively genetically modified and humanized type of donor pig to date (15). The autopsy found that humoral rejection occurred in the transplanted liver accompanied by inflammatory damage, interstitial hemorrhage, and TMA, suggesting that the incorporation of nine human genes from the donor pig did not completely avoid xenograft damage. To a certain extent, this report provided encouragement in that *PERV*-KO/3-KO/9-TG pigs had advantages in inhibiting xenograft rejection and recipients’ coagulation abnormalities.

## 2 Pig Hepatocyte Xenotransplantation

Human hepatocyte transplantation could bridge patients with acute liver injury to liver transplantation until donor livers are available (46), which could even sometimes restore hepatic function. It is noted that significant barriers hinder broader implementation of human hepatocyte transplantation at present, such as limited supply of donor hepatocytes (25), engraftment and repopulation efficiency, cell viability (47), and allogeneic rejection (48). Hepatocytes from pigs are an alternative source that could be unlimited. Besides, transplanted porcine hepatocytes have no more immunogenic than their human counterparts and even could provide protection against human humoral and cellular immune responses (49, 50). There have been preclinical studies on xenotransplantation of hepatocytes from wild-type pigs and genetically-modified pigs into NHPs.

### 2.1 Wild-Type Pig-to-NHP Hepatocyte Xenotransplantation

The first study in experimental preclinical pig-to-NHP hepatocyte xenotransplantation was performed by Nagata et al. in 2007 using hepatocytes from wild-type pigs (51). According to the preliminary clinical experience of fetal hepatocyte allotransplantation, xenogeneic hepatocytes were transplanted into the spleen in three cynomolgus monkeys with normal liver functions, and regular immunosuppressive was used to control the rejection (52). The xenogeneic hepatocytes functioned for more than 80 days, and this was extended to 253 days following re-transplantation. In this study, xenogeneic hepatocyte transplantation did not appear to be affected by the anti-pig antibodies because the vascular endothelium of the liver was not transplanted (53, 54). The isolated xenogeneic hepatocyte transplantation lacks xenogeneic endothelial cells, and the latter are the most vulnerable to humoral immune damage. In this report, because the recipients had a normal liver function at the time of transplantation, it was impossible to predict the extent to which xenogeneic hepatocytes transplantation could restore human liver function.

Porcine hepatocytes microencapsulated in alginatepoly (L-lysine)-alginate microspheres and transplanted intraperitoneally in baboons with fulminant liver failure (FLF) demonstrated effectiveness (20). Capsules or microspheres are designed to protect cells against circulating antibodies and immune or inflammatory cells while allowing nutrients such as oxygen and glucose to diffuse inside the microspheres (55–57). Three of four baboons recovered completely with normal liver function. The remaining baboon developed liver failure but survived 21 days (much longer than the control group). Microencapsulated porcine hepatocytes provided temporary liver function in NHP in the first few days of FLF and increased survival rate, thereby opening new prospects for xenogeneic hepatocyte transplantation.

### 2.2 Genetically Modified Pig-to-NHP Hepatocyte Xenotransplantation

Iwase et al. (50) chose the spleen as the primary site for xenotransplantation in three baboons using hepatocytes from GTKO/hCD46 pigs. And other injection sites were selected according to previous reports in rodents, including lymph nodes (58–61), the subcapsular space of the kidney (62), and subcutaneous fat on the abdominal wall (63). The results were significantly different from Nataga’s report (51), as porcine hepatocytes or infiltrating immune cells were not found in all recipients at any injection sites. The procedures to be optimized in further studies as follows: detect functional viability of the porcine hepatocytes, select a single injection site in one xenotransplantation, measure the cytokine and chemokine responses to hepatocytes, and use the porcine hepatocytes expressing human CD47 and/or HLA-E/G. In conclusion, more basic research is needed before xenogeneic porcine hepatocyte transplantation enters the clinic.

HAR is the most severe rejection when pig livers transplanted into NHPs. Other aspects include severe blood coagulation dysfunction, inflammation and spontaneous internal hemorrhage (32). HAR has been successfully overcome by using GTKO pigs. To mitigate the immunological and physiological molecular incompatibilities between the porcine graft and the human immune system, genetic engineered pigs should be exploited by knocking out more porcine-specific glycan epitopes, overexpression of the hCRPs and coagulation regulatory proteins alone or in combination (15).

## 3 Discussion

In recent decades, basic research of pig-to-NHP LXT has developed rapidly. In the beginning, the leading cause of death of the recipients after LXT was HAR due to incompatibility between species; this led to the longest survival time being only nine days. With the emergence of GTKO and/or hCRPs pig donors, HAR has been basically controlled. The transplanted liver can produce relevant human proteins and maintain several indexes within the regular physiological ranges. However, the subsequent fatal coagulation characterized by severe thrombocytopenia, bleeding, and TMA has become the major obstacle (40).

Due to the use of aprotinin with the effect of inhibiting fibrinolysis, the survival period has been prolonged, and fatal thrombocytopenia no longer occurs in recipients, but blood loss persists. Continuous infusion of coagulation factors can ameliorate the thrombocytopenia and prevent TMA after LXT. The specific mechanism has yet to be elucidated and warrants further investigation (40). Supplementation of exogenous coagulation factors and costimulation blockade after the operation can not only effectively prolong the survival time of recipients but also prevent thrombocytopenia, coagulation disorders, and TMA (41, 42). The potential role of costimulation blockade in prolonging xenograft survival has been verified in other pig organ xenotransplantation models (64, 65).

Heterotopic auxiliary liver transplantation requires native splenectomy, and thus there is no need to excise the native liver; this can not only control postoperative immune rejection and promote the recovery of recipient liver function but also help maintain normal coagulation and anti-coagulation functions in recipients compared to orthotopic LXT (43, 44). The donor liver from the most extensively modified pigs (PERV-KO/3-KO/9-TG) still presents humoral rejection, inflammatory damage, interstitial hemorrhage, and TMA when transplanted into NHPs. These effects suggest that the expression of nine human genes from the donor pig does not entirely avoid xenograft damage by heterotopic auxiliary liver transplantation.

The genetic engineering of pigs renders their cells less immunogenic than their human counterparts (49, 66). The advantages of xenogenetic hepatocyte transplantation over whole liver transplantation are: i) no need to replace all liver functions because the recipient’s own liver will not be removed; ii) being less invasive; iii) allowing the use of encapsulated and/or genetically modified cells; iv) less intense humoral immune response (29, 67), v) low morbidity and higher safety (5); vi) possibility of repeated administration. In contrast to whole organs, which are perfused through the blood vessels of the donor, cellular grafts derive their blood supply by the ingrowth of blood vessels of the recipient, a factor that may be the key to the success of cellular xenografts (68, 69).

Although the remaining issues hinder clinical implementation, donor pigs with optimized genetic modification combinations and highly effective immunosuppressive regimens should be further explored to improve the outcomes of xenogeneic liver and hepatocyte transplantation.

## Author Contributions

XL and HY contributed to select the topic of the manuscript. YW collected relevant references. XL wrote the first draft of the manuscript. HY, YD, and YW wrote sections of the manuscript. All authors contributed to the article and approved the submitted version.

## Conflict of Interest

The authors declare that the research was conducted in the absence of any commercial or financial relationships that could be construed as a potential conflict of interest.

## Publisher’s Note

All claims expressed in this article are solely those of the authors and do not necessarily represent those of their affiliated organizations, or those of the publisher, the editors and the reviewers. Any product that may be evaluated in this article, or claim that may be made by its manufacturer, is not guaranteed or endorsed by the publisher.
